# Prevalence and Associated Factors of Dengue Virus Circulation in the Rural Community, Handeni District in Tanga, Tanzania

**DOI:** 10.1155/2023/5576300

**Published:** 2023-11-08

**Authors:** Debora C. Kajeguka, Francis M. Mponela, Emmanuel Mkumbo, Anna N. Kaaya, Daniel Lasway, Robert D. Kaaya, Michael Alifrangis, Emmanuel Elanga-Ndille, Blandina T. Mmbaga, Reginald Kavishe

**Affiliations:** ^1^Kilimanjaro Christian Medical University College, Moshi, Tanzania; ^2^Pan-African Malaria Vector Control Consortium, Moshi, Tanzania; ^3^Centre for Medical Parasitology, Department Immunology and Microbiology, University of Copenhagen, Copenhagen, Denmark; ^4^Centre for Research in Infectious Diseases (CRID), Yaoundé, Cameroon; ^5^Kilimanjaro Clinical Research Institute, Moshi, Tanzania

## Abstract

Dengue virus is among the most important re-emerging arbovirus that causes global public health attention. Dengue has historically been thought of as an urban disease that frequently occurs in rapidly urbanized settings. However, dengue has become more widespread in rural regions in recent years. Understanding the changing dengue epidemiology in different geographical settings is important for targeted intervention. In Tanzania, dengue fever is not frequently reported because of the poor surveillance infrastructure, underestimation, and a lack of consideration of dengue as a priority. Therefore, the true burden as well as the risk factors for increased transmission has not been fully ascertained, particularly in rural areas. A cross-sectional community-based study was conducted in June 2021, involving a total of 362 participants of all age groups. We investigated the prevalence of acute dengue infection, seroprevalence, and associated factors among the community in three villages of the rural Handeni district. The prevalence of acute dengue infection (based on PCR) was 2.2% (8/362). Dengue-specific IgM and IgG antibodies were detected in 3.3% (12/362) and 5.2% (19/362) of the participants, respectively. Adult participants who were having vegetation around their houses were more likely to be DENV seropositive (AOR = 2.4, CI = 1.88–4.18, *p* value = 0.05). Children living in houses with garbage pit around their households were less likely to be DENV seropositive (AOR = 0.13, CI = 0.03–0.56, *p* value <0.01). DENV continues to circulate in rural Tanzania, causes an alarming situation, and necessitates prompt public health action to enhance vector surveillance and control in rural communities.

## 1. Introduction

Dengue virus (DENV) infection is a mosquito-borne viral disease that contributes to the global health challenge due to its high morbidity and mortality with more than half of the world's population at risk of infection [1, 2]. Each year, up to 400 million people are infected with DENV, and 40000 die from severe DENV infection, while over 80% of infected individuals are having mild symptoms or are asymptomatic [2, 3]. Dengue is caused by four DENV serotypes, DENV 1–4. Dengue can manifest with a wide range of clinical signs, from mild febrile illness to severe plasma leakage that can result in life-threatening shock [4]. The inflammatory cytokine storm and the host response to DENV infection cause the endothelial cells to become more permeable, which causes vascular leakage and contributes to severe dengue [5]. One DENV serotype during primary infection results in long-term immunity against that specific serotype but short time immunity against another serotype, secondary infection with a different serotype increases the likelihood of severe disease [6]. The observed expansion and outbreaks around the globe are facilitated by rapid urbanization, increased travel, globalization, and climate change [7, 8], which affects vector distribution and virus survival.

In Africa, DENV infection is underreported because of the poor surveillance infrastructure and diagnostic capacity, leading to under-estimation of the disease [9, 10], in addition, DENV infection is not considered a priority in many African countries Ministry of Health, Tanzania included. Hence, the true burden of DENV infection is not known. Evidence suggests that DENV infection in African countries' populations may be more widespread than reported [11]. More importantly, DENV infection shares endemicity and similar clinical presentation with chikungunya, malaria, typhoid, and influenza [12], thus leading to difficulty in diagnosis when only clinical diagnosis is used in ruling out the cause of infection.

Dengue vaccine, Denvaxia, is currently approved for use in children aged 9 to 16 who have previously had a laboratory-confirmed DENV infection and who live in dengue-endemic areas. This vaccine is only approved in the United States. Thus, limiting control measures in poor resource countries like Tanzania. Due to the lack of a dengue vaccine or treatment options, treatment is supportive to alleviate the symptoms [13].

Since 2010, Tanzania has experienced reoccurring outbreaks of DENV infection, with the most recent one occurring in 2019 and infecting 6917 people, predominantly in Dar es Salaam and Tanga [14]. The DENV infection season in Tanzania mainly peaks from May to June [14]. Studies conducted during the outbreak showed that the DENV virus primarily spreads in urban areas [15, 16], where *Aedes* mosquitoes thrive because they prefer urban areas [17, 18]. Rapid urbanization is often associated with the emergence and spread of dengue diseases by creating favorable breeding sites such as discarded plastic containers and abandoned car tires [18], as compared to rural areas [19]. More importantly, high population density may increase the risk of infection, although more accessible health systems increase the likelihood of an accurate diagnosis, as well as the availability of tests and more sensitive medical personnel as compared to rural areas.

Many studies on nonmalaria fever have been conducted in Tanzania, but few highlighted those patients with acute DENV infections are often misdiagnosed and more often treated with antimalarial or antibiotics [20–23]. Besides, the consequences of misdiagnosis may lead to economic loss, the development of drug resistance of malaria/bacterial strains (due to over-prescribing of antimalarials and antibiotics), ongoing transmission, and the risk of increased morbidity and mortality. Evidence suggests that several factors are associated with DENV infection transmission, including vegetation, uncovered containers, animal husbandry in the peridomestic environment, stagnant water, and a humid/warm environment favoring the breeding of mosquito vectors [18, 24, 25]. But these studies have been conducted in urban areas thus limiting the epidemiology in the rural community in Tanzania. In other settings, the epidemiology of DENV infection is changing, with reports indicating a global rise in rural infections, particularly in Africa [10, 26]. Conversely, in Gabon, a study suggested minimal DENV infection circulation in rural areas [27], and in Kenya a study reported no evidence of DENV infection circulation in rural communities [28].

The attempts for control are hampered by not understanding the full extent of the epidemiology of DENV infection, especially in rural areas, where it receives little attention. Thus, this study aimed to investigate the prevalence and associated factors and documents the clinical features of DENV infection among the community in a rural area of Tanzania.

## 2. Materials and Methods

### 2.1. Study Setting

This was a cross-sectional study conducted in the Bondo area, Handeni rural, Tanga region, Tanzania. Tanga is located at an elevation of 309 meters above sea level. Its coordinates are 5°22′60″N and 38°34′60″E in DMS (degrees, minutes, and seconds). The area has an annual rainfall of more than 1,212 mm with monthly rainfall peaks in April and May (wet season) of over 470 mm, and September to October (short rains) with over 250 mm [29]. Tanga is endemic to malaria with a prevalence of 14.6% [30], while the prevalence of fever is 14.8% [31], which could generally be attributed to viral, bacterial, or malaria infection. Most DENV transmission occurs during the rainy season as a result of increased *Aedes* vector abundance. Three villages have been purposively selected, namely, Bondo, Kwamgwe, and Kwadoya.

### 2.2. Sample Size and Inclusion Criteria

Simple random sampling was used to identify participants that met the inclusion criteria until the desired sample size was reached. The minimum sample size for this study was calculated using the following formula *n* = *z*^2^*pq*/*d*^2^. In this equation, *n* is the sample size, *z* is the value of the standard normal distribution at the 5% level (1.96), *p* is the prevalence, *q* = 1 − *p*, and *d* is the precision level (0.05). The prevalence of acute DENV infection is 38.2% [32]. Sample size = 1.962 × 0.382 × (1 − 0.38)/(0.05)^2^. The sample size was 362 for the detection of DENV infection. The study included children (2–17 years old), and adults (18–70 years old) who volunteered to participate in the study, and who had lived in the area for at least a year. We excluded individuals with any signs of severe illness due to other causes other than arboviral diseases and those who were mentally unfit.

### 2.3. Recruitment and Interview

Community members and village leaders were involved before recruiting study participants. During recruiting and enrollment, randomly chosen participants were invited to the neighborhood dispensary or schools. Meetings were organized wherein study staff explained the purpose of the study and answered any questions in an open forum. Completing questionnaires with an interviewer started immediately after participants have consented to participate in the study. Participants were enrolled only after verification of the potential inclusion criteria. After consenting, a structured Swahili questionnaire was used to collect information among the study population. The main contents of the questionnaire included general demographic characteristics (age, gender, marital status, education, average household (HH) income, and occupation). It also included social and personal life activities, such as travel history, outdoor sports, and any social events. Moreover, the questionnaire presented questions related to the prevention and control of mosquitoes. Also, the questionnaire included environmental sanitation (presence of garbage pits or vegetation near the house), housing characteristics, such as the building structure and roofing, and living conditions (average numbers of people sharing a room). Lastly, we collected information regarding the history of fever or illness for the past 24 hours and blood sample collection, and laboratory investigation (Supplementary File 1).

### 2.4. Blood Sample Collection and Serological Testing of DENV

Approximately 0.4 ml of blood samples were drawn from the participants.

A few drops of blood samples were used for the detection of DENV-specific IgM and IgG antibodies using on-site rapid dengue immunoglobulins M/G (IgM/IgG) (CTK Biotech, USA) according to the manufacturer's instructions. Briefly, blood samples were placed in a test kit, buffer was added, and waited for approximately 15 minutes before providing final results. Results interpretation for all rapid tests was done visually by the naked eye. In cases where the final results were ambiguous, a second confirmation from another laboratory technologist was requested. Two laboratory technologists who are accredited and licensed by the proper authorities conducted all of the testing. The remaining blood samples were placed in EDTA tubes and stored temporarily in liquid nitrogen before transport to the Kilimanjaro Christian Medical University College (KCMUCo) laboratory for storage at −80°C for further analysis.

### 2.5. RNA Extraction

Viral RNAs were extracted from human whole blood using the Qiagen RNA Blood Mini Kits according to the manufacturer's instructions (Qiagen, Hilton Germany). Briefly, an aliquot of 30 *µ*L of the blood sample was lysed and then homogenized using QIAshreader spin Coulmn. The samples were applied to the spin column. Total RNA binds to the QIAamp membrane. Pure RNA was eluted in a total of 30 to 100 *µ*L of RNA-free water and stored at −20°C ready for PCR.

### 2.6. Detection of DENV by RT-PCR

The Aridia Zika/Dengue/CHIK real-time PCR test, in a one-step format, was used to detect the DENV infection (CTK Biotech, Inc., Poway, California, USA). The lyophilized dengue-positive control was rehydrated in 100 *μ*L of supplied PCR-grade water. A total of 5 *μ*L of each extracted RNA, negative control, and positive control were pipetted in the respective wells in the plate. The amplification reaction was performed in an AriaMx Agilent PCR machine (Agilent, Santa Clara, California, USA). The thermocycler conditions started with reverse transcription at 45°C for 15 minutes and an initial denaturation at 95°C for 2 minutes, followed by 45 cycles of denaturation at 95°C for 10 seconds and annealing/extension at 60°C for 50 seconds. Fluorogenic data analysis of the samples and controls was performed by the real-time PCR thermocycler software, according to the manufacturer´s instructions.

### 2.7. Data Analysis

The data collected were checked for completeness, cleaned, and entered into Statistical Package for Social Sciences (SPSS) version 26 for analysis. socioeconomic status (SES) proxy was created using principal component analysis (PCA) to investigate the independent effects of wealth indices on disease status [33]. Categorical variables were summarized into frequency and percentage while the continuous variable was summarized by median and Interquartile range. The chi-square (*χ*^2^)/Fischer exact test was used to determine the association between independent factors and the outcome variables.

To investigate the association between dengue virus circulation and independent factors, bivariate analysis was first conducted for each potentially explanatory factor that was independently associated with DENV seropositivity. Variables with a *p* value <0.1, were considered in the multivariable analysis. Multivariable logistic regression was used to estimate the adjusted effect of factors associated with DENV. *p* values of ≤0.05 were recognized as significant.

### 2.8. Ethical Considerations

The approval was sought from the Kilimanjaro Christian Medical University College Research and Ethics Review Committee (CRERC) with certificate number 2492 and the National Institute of Medical Research with certificate number NIMR/HQ/R.8a/Vol.IX/3651. Permission to conduct this study was granted by the district medical officer (DMO) of the Handeni district and the village leaders. Written informed consent was obtained from all participants. Assent involved participants in the age range of 2 to 17 years, who were legally not able to consent by themselves. Parents or guardians consented on behalf of participants who were under 18 years.

The participant who was not able to read or write an impartial witness signed on their behalf. Confidentiality was adhered to where participant information was available to the researcher only. The study was conducted by following Good Clinical and Laboratory Practices.

## 3. Results

### 3.1. Social-Demographic Characteristics of the Participants

The study included 362 participants, the majority were from Bondo village 144 (39.8%). The median age was 28.3 (IQR: 25.9–30.7). Kwadoya had a significantly higher proportion of participants with ≥18 years as compared to Bondo or Kwamgwe villages (*χ*^2^ = 13.07, *p* = 0.01). Bondo had a significantly higher proportion of participants with primary education as compared to Kwadoya or Kwamgwe (*χ*^2^ = 11.55, *p* = 0.05). Most participants were farmers from Bondo (*χ*^2^ = 8.88, *p* = 0.008). Lastly, significantly higher proportion participants were having a monthly average income below 85 USD (*χ*^2^1.35, *p* = 0.5), Table 1.

### 3.2. Prevalence of DENV-IgM/IgG and Acute DENV Infection

Generally, DENV-specific IgM antibodies were detected in 3.3% (12/362) of the participants. Bondo village recorded a higher prevalence of DENV IgM at 6.3% (9/144), followed by Kwamgwe at 1.6% (2/125), and lastly Kwadoya at 1.1% (1/93), (Fischer exact test = 5.5, *p* = 0.03). There were no differences in IgM antibody seropositivity among participants with different age groups, sex, and occupation (*p* > 0.05). DENV-specific IgG antibodies were detected in 5.2% (19/362) of the participants. Bondo village recorded the highest prevalence of DENV IgG at 5.6% (8/144), followed by Kwadoya at 5.4% (5/93), and lastly Kwamgwe at 4.8% (6/125), (*χ*^2^ = 0.08, *p* > 0.05). Also, there were no differences in IgG antibody seropositivity among participants with different age groups, sex, and occupation (*p* > 0.05). Results show that 85.7.0% (24) of DENV seropositive participants had no history of traveling outside the study area or region.

Acute DENV infection (as detected by PCR) was recorded in 2.2% (8/362).

The age range, 6 to 17 years, was more affected by 4.7% (6/129) compared to another age group (Fischer exact test = 4.41, *p* = 0.1), though there was no significant difference. Bondo recorded the highest prevalence of acute DENV infection at 3.5% (5/144) compared to Kwamgwe at 1.6% (2/125) and Kwadoya at 1.1% (1/93), (Fischer exact test = 1.49, *p* > 0.05). Likewise, there were no differences in DENV infection between males and females as well as occupation (*p* > 0.05). Only one participant was positive by IgM and PCR test, Table 2. Results show that 75.0% (5) of DENV PCR-positive participants had no history of traveling outside the study area or region.

### 3.3. Common Symptoms among Participants with Positive PCR, IgM, and/or IgG Results

Generally, commonly reported clinical signs and symptoms among participants include headache 31.9% (123), abdominal pain 17.6% (68), muscle pain 11.1% (43), arthritis/tiredness 10.9% (42), colds 7.8% (30), chills 6.2% (24), sweating 5.4% (21), nausea 4.7% (18), persistent vomit 3.1% (12), and diarrhea 1.3% (5), Table 3. Among DENV IgM-positive participants, 6.5% (8) were having a headache, 7.0% (3) had muscle pain, 5.6% (1) had nausea, 4.2% (1) had chills, and 6.7% (2) had cold. Among those with dengue warning signs, 2.9% (2) were having abdominal pain, 8.3 (1) were having persistent vomiting, and 7.1% (3) were having arthritis/tiredness. Among DENV IgG-positive participants, 0.8% (1) had a headache, 2.3% (1) had muscle pain, and 4.2% (1) had chills. Among those with dengue warning signs, 1.5% (1) had abdominal pain, and 4.8% (2) were having arthritis/tiredness. Only 1.6% (2), 4.2% (1), 10.0% (3), 2.9% (2), and 2.4% (1) were infected with DENV infection headache, chills, cold, abdominal pain, and tiredness/arthritis, respectively, Table 3.

### 3.4. Factors Associated with DENV Seropositivity among Adults

In bivariate analysis, only wells/stagnant water and vegetation were significant predictors of DENV seropositivity (*p* set at <0.1) (Table 4). The significant findings of the bivariate analysis were further analyzed in the final model of multivariate analysis using logistic regression. Participants who had wells/stagnant water around their houses were less likely to be DENV seropositive (AOR = 0.01, CI = 0.02–0.08, *p* value <0.001). Participants who were having vegetation around their houses were more likely to be DENV seropositive (AOR = 2.4, CI = 1.88–4.18, *p* value = 0.05).

### 3.5. Factors Associated with DENV Seropositivity among Children

In bivariate analysis, only average people sharing a room, garbage pit, vegetation, and roofing were significant predictors of DENV seropositivity (*p* set at <0.1) (Table 5). The significant findings of the bivariate analysis were further analyzed in the final model of multivariate analysis using logistic regression. The final model of DENV seropositivity showed that two potential independent factors were significantly associated with DENV seropositivity: participants who had garbage pit around their houses were less likely to be DENV seropositive (AOR = 0.13, CI = 0.03–0.56, *p* value <0.01). Contrary to participants whose houses had grass or tile roofs, those with corrugated iron sheets had a higher likelihood of having DENV seropositive results (AOR = 3.90, CI = 0.94–16.11, *p* value = 0.05).

## 4. Discussion

The study investigated the prevalence of acute DENV infection and seroprevalence as well as associated factors of DENV seropositivity among the community in the rural Handeni district in Tanga. Generally, we found that 3.3% and 5.2% of participants were seropositive to DENV IgM and IgG, respectively. Moreover, Bondo village had a higher IgM and IgG seroprevalence compared to other villages, although not significantly. Our findings are lower than the study conducted in eight districts of Tanzania which reported a prevalence of 28.6% IgG-positive in the northeastern zone represented by Kilindi which is close to Kwamgwe [34]. Lower results in our study could partly be attributed to the reason that our study was conducted in a community while the other study was conducted in hospital settings. Acute DENV infection was detected in 2.2% of participants, but there was no statistically significant difference between villages. Previous studies conducted in the same settings reported that none of the study participants were dengue-positive during the study period [22]. This implies that DENV infection continues to emerge in recent years. Our findings may justify the continued and persistent circulation of DENV infection in Tanzania. Also, our findings explain the ongoing autochthonous transmission as evidenced by our data showing that all participants who tested positive for DENV infection did not travel outside the study area or region where they could contract the virus.

The study shows that there is no difference in DENV infection between villages, this suggests that the risk of being infected by DENV is relatively homogenous within the populations [35], and thus villages have relatively similar characteristics. It has been reported elsewhere that DENV infection has evolved from small outbreaks previously seen to large outbreaks experienced recently in 2019 [14, 15]. This implies that DENV infection is likely spreading and circulating to a larger extent and perhaps in other parts of Tanzania such as rural areas which goes unnoticed. Regarding the symptoms of DENV infection, the participant complained mainly about headache, abdominal pain, muscle pain, and arthralgia/tiredness. The same observations were reported in a study conducted in Dar es Salaam which reported a higher number of DENV-infected patients who complained about headaches and muscle pain. One possible explanation for this discrepancy is because the study in Dar es Salam was conducted in a hospital setting [36].

We conducted logistic regression differently in adults and children. Our results showed that adult participants with the presence of wells/stagnant water around the home were less likely to be DENV seropositive. This may be explained by the fact that *Aedes* mosquitoes do not breed in wells/stagnant water around houses. However, other studies in Tanzania reported that stagnant water is associated with DENV seropositivity [34]. Further studies should be done to reveal this.

In the present study, adult participants who had vegetation around their HH were likely to be DENV seropositive compared to others. It has been reported that vegetation is associated with higher *Aedes* density as vegetation provides a more suitable resting habitat [37, 38]. It favors breeding sites close to dense vegetation including plantations which are linked to an increased risk of exposure for rural workers such as those in rubber and palm oil plantations, but it is also found to be established abundantly in urban areas [39, 40]. Previous studies conducted elsewhere displayed an association between greenery and the number of DENV infections with the perception that mosquitoes are abundant in highly vegetated areas [41, 42].

Similar to adults, several factors have been linked to DENV seropositivity in children. Our study reports that sharing a room with more than 3 people per HH is associated with DENV seropositivity. The same results have been long reported from studies conducted in Brazil [43] and Venezuela [44], indicating that more people in a room or crowded living is the most likely factor that increases DENV transmission. This may be relevant given the fact that *Aedes* mosquito bites during the day and the majority of parents do not cover their children with bednet when they sleep during the day. Participants with garbage pits were less likely to be DENV seropositive compared to those who did not. A different scenario has been reported in an urban area whereby areas with a high population density, presence of garbage pits, or solid waste disposal facilities are poor, and the open container may provide conducive breeding sites for *Aedes* mosquitoes [34, 45]. In contrast to the urban area, the reason for our observed results could be in the rural areas the frequent disposal of HH plastic wastes or containers is uncommon. In our study, participants' houses with corrugated iron sheets were more likely to be DENV seropositive compared to those with grass or tiles. This could be explained by the fact that houses with corrugated iron sheet tend to be warmer than other houses, thus affecting mosquito incubation and speeding up the transmission of the virus [46].

### 4.1. Strength and Limitation

The community-based study was more representative of the general population compared to other studies, which included febrile patients seeking healthcare. The study could not establish a causal relationship between independent and dependent variables because of the cross-sectional nature of the study. A cohort study is recommended. In clinical practice, it is difficult to directly use IgM-based results to make a clinical decision. Detection of IgM antibodies in a patient is often deciphered as an indicator of recent and sometimes acute infections, however, caution should be considered due to the reason that false-positive IgM results are common, as a result of cross-reactivity with IgM antibodies to other, closely related arbovirus such as yellow fever and West Nile [47, 48]. IgM antibodies may remain detectable up to 3 months and more [49]. However, the rapid test used in this study has a sensitivity and specificity of 96.6% and 98.1%, respectively [50].

## 5. Conclusions

DENV continues to circulate in rural Tanzania and causes an alarming situation and there is an urgent need to develop targeted interventions and strengthen the surveillance system to address the issue. The identified factors of DENV seropositivity in this study could contribute to directing more targeted integrated preventive and control measures. In addition, community engagement is paramount in vector control. These findings will help the Tanzania Ministry of Health plan for effective integrated control programs in rural communities.

## Figures and Tables

**Table 1:** Socio-demographic characteristic of participants (*n* = 362).

Variables		Bondo % (*n*)	Kwadoya % (*n*)	Kwamgwe % (*n*)
Age	<5 Years	10.4 (15)	8.6 (8)	18.4 (23)
	6 to 17 Years	34.7 (50)	29.0 (27)	41.6 (52)
	≥18 Years	54.9 (79)	62.4 (58)	40.0 (59)

Sex	Male	39.6 (57)	45.2 (42)	43.2 (54)
	Female	60.4 (87)	54.8 (51)	56.8 (71)

Education level	Primary	66.7 (96)	60.2 (56)	49.6 (62)
	Secondary	2.8 (4)	4.3 (4)	7.2 (9)
	Tertiary	1.4 (2)	1.1 (1)	0 (0)
	Not gone to school/child	29.2 (42)	34.4 (32)	43.2 (54)

Occupation	Farmer	88.9 (128)	95.7 (89)	86.4 (108)
	Business/self-employed	5.6 (8)	3.2 (3)	10.4 (13)
	Employed	5.6 (8)	1.1 (1)	3.2 (4)

Average HH income per month	<85 USD/-	91.7 (132)	94.6 (88)	90.4 (113)
	85–420 USD	8.3 (12)	5.4 (5)	9.6 (12)

Total		39.8 (144)	25.7 (93)	34.5 (125)

HH: household.

**Table 2:** Seroprevalence and prevalence of DENV infection in the participants (*N* = 362).

Variables		*N*	DENV IgM (rapid test) positive % (*n*)	DENV IgG (rapid test) positive % (*n*)	DENV-PCR positive % (*n*)
Total		362	3.3 (12)	5.2 (19)	2.2 (8)

Age (years)	≤5	46	4.3 (2)	10.9 (5)	0
	6 to 17	129	2.3 (3)	3.9 (5)	4.7 (6)
	≥18	187	3.7 (7)	4.8 (9)	1.1 (2)
			*p* > 0.05	*p* > 0.05	*p* = 0.1

Sex	Male	153	4.3 (9)	5.2 (8)	1.3 (2)
	Female	209	2.0 (3)	5.3 (11)	2.9 (6)
			*p* > 0.05	*p* > 0.05	*p* > 0.05

Occupation	Farmer	325	3.1 (10)	5.5 (18)	1.8 (6)
	Business/self-employed	24	4.2 (1)	4.2 (1)	4.2 (1)
	Employed	13	7.7 (1)	0 (0.0)	7.7 (1)
			*p* > 0.05	*p* > 0.05	*p* > 0.05

Village	Bondo	144	6.3 (9)	5.6 (8)	3.5 (5)
	Kwadoya	93	1.1 (1)	5.4 (5)	1.1 (1)
	Kwamgwe	125	1.6 (2)	4.8 (6)	1.6 (2)
			*p* = 0.03	*p* > 0.05	*p* > 0.05

**Table 3:** Signs and symptoms among dengue and nondengue-positive participants (*N* = 362).

Variables		% (*n*)	DENV IgM		DENV IgG		DENV-PCR	
			Positive % (*n*)	Negative % (*n*)	Positive % (*n*)	Negative % (*n*)	Positive % (*n*)	Negative % (*n*)
Symptoms and signs	Headache	31.9 (123)	6.5 (8)	93.5 (115)	0.8 (1)	99.2 (122)	1.6 (2)	98.4 (121)
	Muscle pain	11.1(43)	7.0 (3)	93.0 (40)	2.3 (1)	97.7 (42)	—	100 (43)
	Nausea	4.7 (18)	5.6 (1)	94.4 (17)	0 (0)	100 (18)	—	100 (18)
	Diarrhea	1.3 (5)	—	100 (5)	0 (0)	100 (5)	—	100 (5)
	Chill	6.2 (24)	4.2 (1)	95.8 (23)	4.2 (1)	95.8 (23)	4.2 (1)	95.8 (23)
	Cold	7.8 (30)	6.7 (2)	93.3 (28)	0 (0)	100 (30)	10.0 (3)	90.0 (27)
	Sweating	5.4 (21)	4.8 (1)	95.2 (20)	0 (0)	100 (21)	—	100 (2)

Dengue warning signs	Abdominal pain	17.6 (68)	2.9 (2)	97.1 (66)	1.5 (1)	98.5 (67)	2.9 (2)	97.1 (66)
	Persistent vomiting	3.1 (12)	8.3 (1)	91.7 (11)	0 (0)	100 (12)	—	100 (12)
	Tiredness/arthritis	10.9 (42)	7.1 (3)	92.9 (39)	4.8 (2)	95.2 (40)	2.4 (1)	97.6 (41)

**Table 4:** Factors associated with DENV seropositivity (adults).

Variables		% (*n*)	COR (95% CI)	*p* value	AOR (95% CI)	*p* value
Sex	Male	40.1 (75)	Reference		—	—
	Female	59.9 (112)	0.88 (0.30–2.25)	0.8		

Marital status	Married	69.5 (130)	Reference		—	—
	Single	20.9 (39)	1.88 (0.40–8.79)	0.4		
	Divorced	9.6 (18)	0.81 (0.16–3.97)	0.7		

Income	<USD 85/-	90.4 (169)	Reference		—	—
	USD 85/- to 400	9.6 (18)	0.60 (0.07–4.48)	0.6		

^*∗*^SES	High	4.3 (8)	Reference		—	—
	Low	80.2 (150)	1.50 (0.17–13.20)	0.7		
	Medium	15.5 (29)	1.92 (0.15–24.46)	0.6		

Average people sharing a room	Up to 2	61.5 (115)	Reference		—	—
	≥3	38.5 (72)	1.67 (0.59–4.67)	0.3		

Travel history (past 2 weeks)	No	90.4 (169)	Reference		—	—
	Yes	9.6 (18)	0.72 (0.15–3.46)	0.6		

Wells/stagnant water (within 200 meters)	No	95.2 (178)	Reference			
	Yes	4.8 (9)	0.10 (0.03–0.08)	<0.001	0.01 (0.02–0.08)	<0.001

Garbage pit (within 200 meters)	No	31.6 (59)	Reference		—	—
	Yes	68.4 (128)	0.70 (0.21–2.27)	0.5		

Vegetation (within 200 meters)	No	61.5 (115)	Reference			
	Yes	38.5 (72)	3.96 (1.59–11.94)	0.01	2.4 (1.88–4.18)	0.05

House building structure	Wood	18.7 (35)	Reference		—	—
	Mud	52.9 (99)	1.08 (0.10–11.31)	0.9		
	Blocks	28.3 (53)	4.0 (0.41–39.55)	0.2		

^*∗*^Calculated based on the wealth asset index. SES: socioeconomic status.

**Table 5:** Factors associated with DENV seropositivity (children).

Variables		% (*n*)	COR (95% CI)	*p* value	AOR (95% CI)	*p* value
Sex	Male	44.6 (78)	Reference		—	—
	Female	55.4 (97)	0.81 (0.27–2.39)	0.7		

Average people sharing a room	Up to 2	54.9 (96)	Reference			
	≥3	45.1 (79)	2.63 (0.86–8.06)	0.08	2.57 (0.71–9.23)	0.1

Travel history (past 2 weeks)	No	89.7 (157)	Reference		—	—
	Yes	10.3 (18)	0.41 (0.10–1.63)	0.2		

Wells/stagnant water (within 200 meters)	No	17.7 (31)	Reference		—	—
	Yes	82.3 (144)	0.69 (0.14–3.24)	0.6		

Garbage pit (within 200 meters)	No	49.1 (86)	Reference			
	Yes	50.9 (89)	0.23 (0.06–0.85)	0.02	0.13 (0.03–0.56)	<0.01

Vegetation (within 200 meters)	No	43.4 (76)	Reference			
	Yes	56.6 (99)	2.25 (0.68–7.36)	0.1	3.42 (0.90–13.05)	0.07

House building structure	Wood	15.4 (27)	Reference		—	—
	Mud	63.4 (111)	0.66 (0.13–3.14)	0.6		
	Blocks	21.1 (37)	2.88 (0.24–33.51)	0.3		

Roofing	Tiles	13.7 (24)	Reference			
	Grass roof	17.7 (31)	1.68 (0.37–7.54)	0.4	1.09 (0.20–5.74)	0.9
	Corrugated iron sheet	68.6 (120)	4.56 (1.35–15.31)	0.01	3.90 (0.94–16.11)	0.05

## Data Availability

The dataset of the current study will be available from the corresponding author upon reasonable request.

## Supplementary Materials

Supplementary Materials Supplementary File 1: Questionnaire. Part one: demographic characteristics. Part two: bednet use, protection against mosquito bite and housing characteristics. Part three: travel history. Part four: illness. Part five: laboratory investigations.
