# Supplementary material for: Prevalence and Associated Factors of Dengue Virus Circulation in the Rural Community, Handeni District in Tanga, Tanzania
Source: J Trop Med. 2023 Nov 8;2023:5576300. doi: 10.1155/2023/5576300 (PMC10651340; doi:10.1155/2023/5576300)
Supplement: Supplementary Materials — Supplementary File 1: Questionnaire. Part one: demographic characteristics. Part two: bednet use, protection against mosquito bite and housing characteristics. Part three: travel history. Part four: illness. Part five: laboratory investigations. [file 5576300.f1.docx]

**QUESTIONNAIRE**

**SABIOT PROJECT, TANZANIA**

**KILIMANJARO CHRISTIAN MEDICAL UNIVERSITY COLLEGE**

**AUTHOR: DEBORA KAJEGUKA**

**PART ONE: DEMOGRAPHIC CHARACTERISTICS**

| **S/N** | **VARIABLES** | **RESPONSE** |
| --- | --- | --- |
| 1 | Sex | 1. Male 2. Female |
| 2 | Age (in years) |  |
| 3 | What is the highest level of education you have attained? | 1. A Child (Still in primary school/Kindergarten) 2. No formal education 3. Primary education 4. Secondary education 5. Higher education |
| 4 | Marital status | 1. Single 2. Married 3. Divorced 4. Widowed 5. Others……………………….. |

**PART TWO: BEDNET USE, PROTECTION AGAINST MOSQUITO BITE AND HOUSING CHARACTERISTICS**

| **S/N** | **VARIABLES** | **RESPONSE** |
| --- | --- | --- |
| 1 | Color of your bednet | 1. White 2. Blue 3. No bednet |
| 2 | IF the answer is “NO BEDNET “above  Reason for not having net | 1. I Do not like 2. I have Financial problem 3. It was finish 4. I was Not available during distribution 5. Not yet given 6. Other…………………….. |
| 3 | Source of net | 1. Antenatal visit (hospital) 2. Free distribution 3. It was a Gift 4. I Purchased |
| 4 | BedNet brand | 1. Olyset (Permethrin treated) 2. Olyset Plus (Permethrin+PBO) 3. Safinet 4. Others (mention)…….……………………. |
| 5 | Did you/you child sleep under the bed last night? | 1. Yes 2. No |
| 6 | If Answer is “YES”  Do you encounter problem during the use Insecticide treated Nets, Please mention problems | ……………………………………………………………….  ……………………………………………………………….  ………………………………………………………………. |
| 7 | Period of bednet usage | 1. Dry season 2. Rainy season 3. Throughout the year |
| 8 | Reasons for sleeping under the net | 1. Avoid malaria 2. Avoid dengue or chikungunya 3. Protect myself 4. Protect child 5. Protect myself and child 6. Others, mention………………………. |
| 9 | Average number of person sleeping in one room | 1. One 2. Two 3. More than three |
| 10 | List any other way you use to protect yourself/you child from mosquito bite  (specify) |  |
| 11 | Number of residence per household | 1. One 2. Two 3. Three 4. More that three |
| 12 | Monthly average per capital income | 1. <200,000 TZS 2. 200,000-1 million 3. More than 1 Million |
| 13 | Housing Building structure | 1. Bricks (made of mud) 2. Blocks (Made of cement) 3. Makuti and Mud 4. Others specify…………………………………… |
| 14 | House roofing | 1. Grass roof 2. Corrugated iron sheet 3. Tiles 4. Others specify………………. |
| 15 | Is there any Garbage collection pit within 200m around your home | 1. Yes 2. No |
| 16 | Is there any pond/well within 200m around your home | 1. Yes 2. No |
| 17 | Is there wells/stagnant water | 1. Yes 2. No |
| 18 | Is there vegetation around housed (Within 200m) | 1. Yes 2. No |

**PART THREE: TRAVEL HISTORY**

| **S/N** | **VARIABBLE** | **RSPONSE** |
| --- | --- | --- |
| 1 | Have you travelled outside this village for more than two weeks ago  (Must be outside Region) | 1. Yes 2. No |
| 2 | If the answer is “YES” (Mention the region(s)) |  |
| 3 | If “Yes” Did you use bednet while you were away? | 1. Yes 2. No |
| 4 | Have you travelled outside this village for social events (More than two weeks ago) | 1. Yes 2. No |
| 5 | Have you participated in outdoor sports | 1. Yes 2. No |
| 6 | If YES, Do you do the following? | 1. Camping 2. Religious events 3. Others, mention_________________ |

**PART FOUR: ILLNESS**

| **S/N** | **VARIABLE** | **RESPONSE** |
| --- | --- | --- |
| **1** | Body Temperature Measurements (in ^o^C) | **………………………………………** |
| 2 | Have you been ill for the past two weeks? | 1. Yes 2. No |
| 3 | If the answer is “YES”, what were you suffering? (TICK ALL SIGNS AND SYMPTOMS THAT APPLYS) | 1. Fever 2. Headache 3. Abdomen pain 4. Muscle pain 5. Chills 6. Tiredness 7. Night sweats 8. Shivering 9. Diarrhea 10. Nausea 11. Vomiting 12. Bleeding |
| 4 | Do you currently have any illness? | 1. Yes 2. No |
| 5 | If the answer is “ YES”, what are the signs and symptoms? (LIST) |  |
| **PART FIVE: LABORATORY INVESTIGATIONS** | |  |
| 1 | Blood sample taken? | 1. Yes 2. No |
| 2 | THICK and THIN smear prepared | 1. Yes 2. No |
| 3 | Filter paper/Blood spots prepared | 1. Yes 2. No |
| 4 | mRDT | 1. Positive 2. Negative |
| 5 | Dengue IgM | 1. Positive 2. Negative |
| 6 | Dengue IgG | 1. Positive 2. Negative |
| 7 | Chikungunya IgM | 1. Positive 2. Negative |
| 8 | Hb level [ grams per deciliter (g/dl)] | ……………………………… |
